# Characteristics of brittle cornea syndrome by multimodal imaging modalities: a case report

**DOI:** 10.1186/s12886-023-03123-9

**Published:** 2023-09-15

**Authors:** Huixian Wang, Xu Zhang, Xiaowei Gao, Wenjing Li

**Affiliations:** Xinjiang 474 Hospital (the former 474 Hospital of the People’s Liberation Army), Beijing Road 754, Urimqi, 830000 Xinjiang Autonomous Region China

**Keywords:** Brittle cornea syndrome, Blue sclera, Keratoglobus, Corneal densitometry, Case report

## Abstract

**Background:**

A report of a Brittle cornea syndrome (BCS) case with bluish scleral discoloration, keratoglobus, and myopia based on multimodal imaging modalities including in vivo confocal microscopy (IVCM), high-definition optical coherence tomography (HD-OCT) and scheimpflug corneal densitometry analysis.

**Case presentation:**

A 36-year-old Chinese female patient presented with significant bluish discoloration of the sclera in both eyes, extreme corneal thinning with increased corneal curvature, increased central corneal densitometry, and nystagmus. She also had scoliosis, severe osteoporosis, and thyroid disease.

**Conclusions:**

Timely diagnosis, early detection, and detailed follow-up are essential for BCS. There has been no report of a BCS evaluation performed by IVCM and corneal densitometry methods thus far in the literature. Furthermore, multimodal imaging can offer a more comprehensive view of BCS and contribute to a deeper understanding of the disease. Interestingly, this is a rare case of BCS in an adult with good vision, an intact cornea, and nystagmus.

## Background

BCS is an autosomal recessive condition characterized by significant corneal fragility and thinning [1, 2]. Other ocular characteristics include: blue sclera, keratoconus, keratoglobus, and an extreme degree of myopia. Extraocular manifestations include deafness, scoliosis, mild joint hypermobility, and hip dysplasia [3]. The majority of these extraocular characteristics were present in this patient, including consanguineous parentage, scoliosis, severe osteoporosis, and thyroid dysfunction. The thinness of the cornea places the patient at an increased risk of spontaneous rupture. These limit the patient’s ability to receive additional examinations, thus preventing us from obtaining a comprehensive picture of the patient's ocular condition. To our knowledge, there are no previous cases of BCS that have been evaluated by in vivo confocal microscopy (IVCM) and corneal densitometry. This is a rare case of BCS in an adult with good vision, an intact cornea, and nystagmus.

## Case presentation

**Fig. 1:**
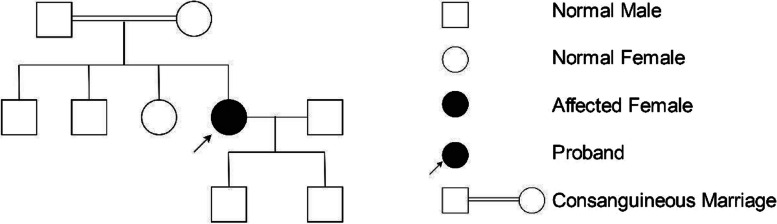
Pedigree chart

**Fig. 2:**
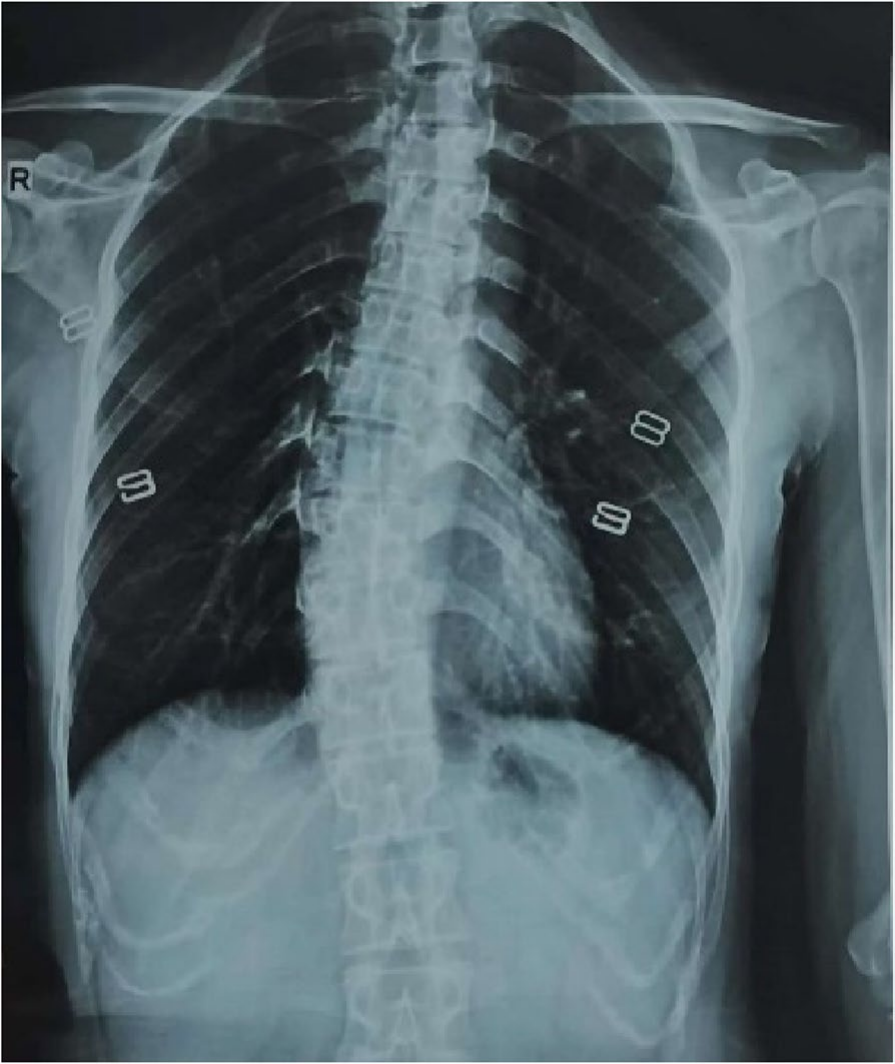
Spine X-Ray: scoliosis of approximately 20°

**Fig. 3:**
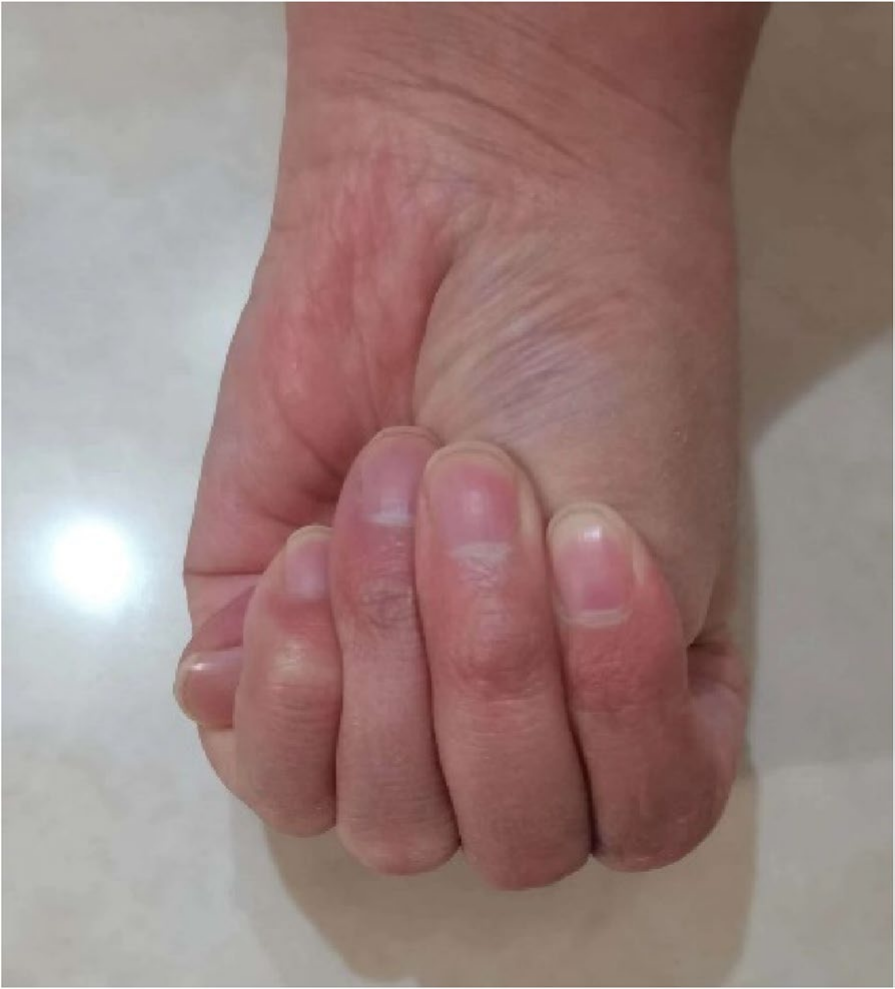
Thumb sign in which thumb is protruding out and is visible medial to the little finger

**Fig. 4:**
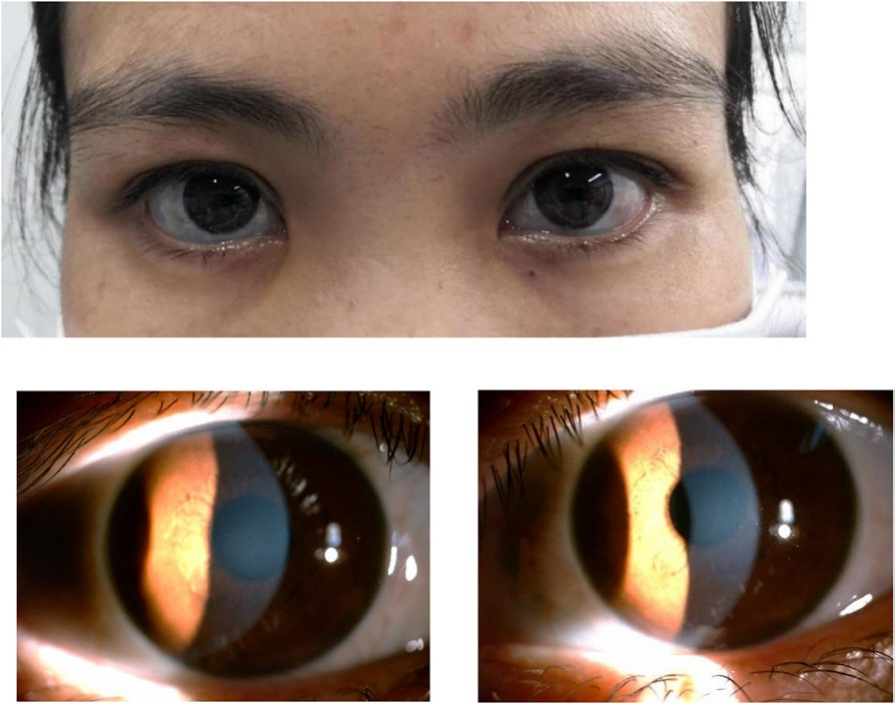
Slit lamp microscopy showed central corneal transparency appears to have decreased

**Fig. 5:**
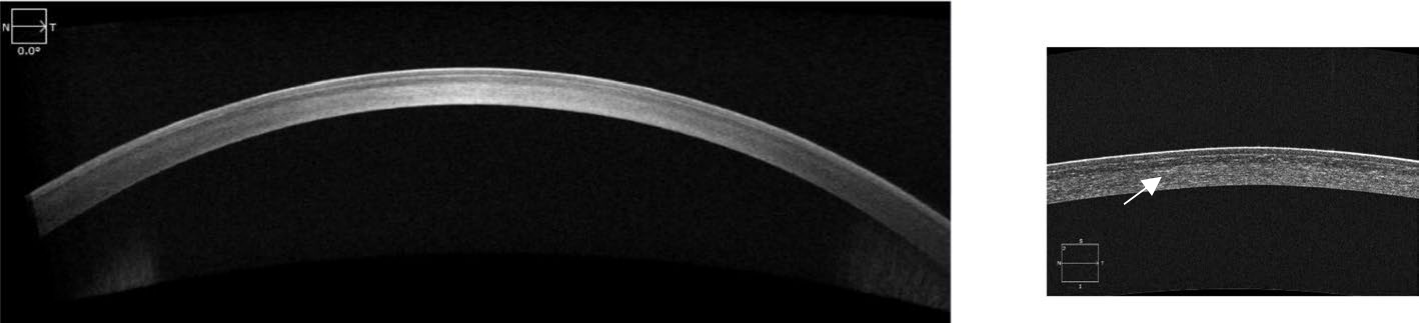
HD-OCT of the right eye showed thinning of the whole cornea and heterogeneous and mildly elevated stromal layer density by the arrow

**Fig. 6:**
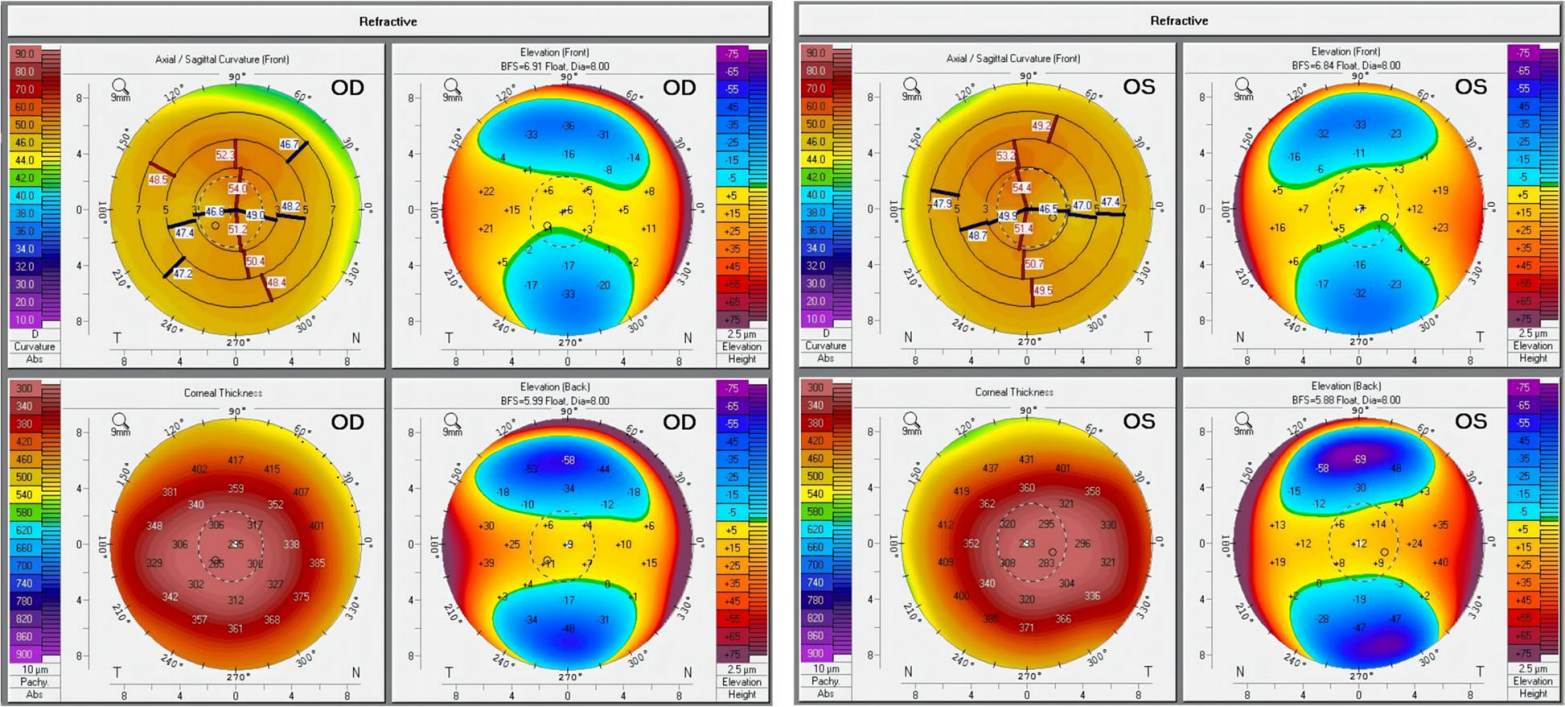
Pentacam-refractive maps of the right and left eyes of case

**Fig. 7:**
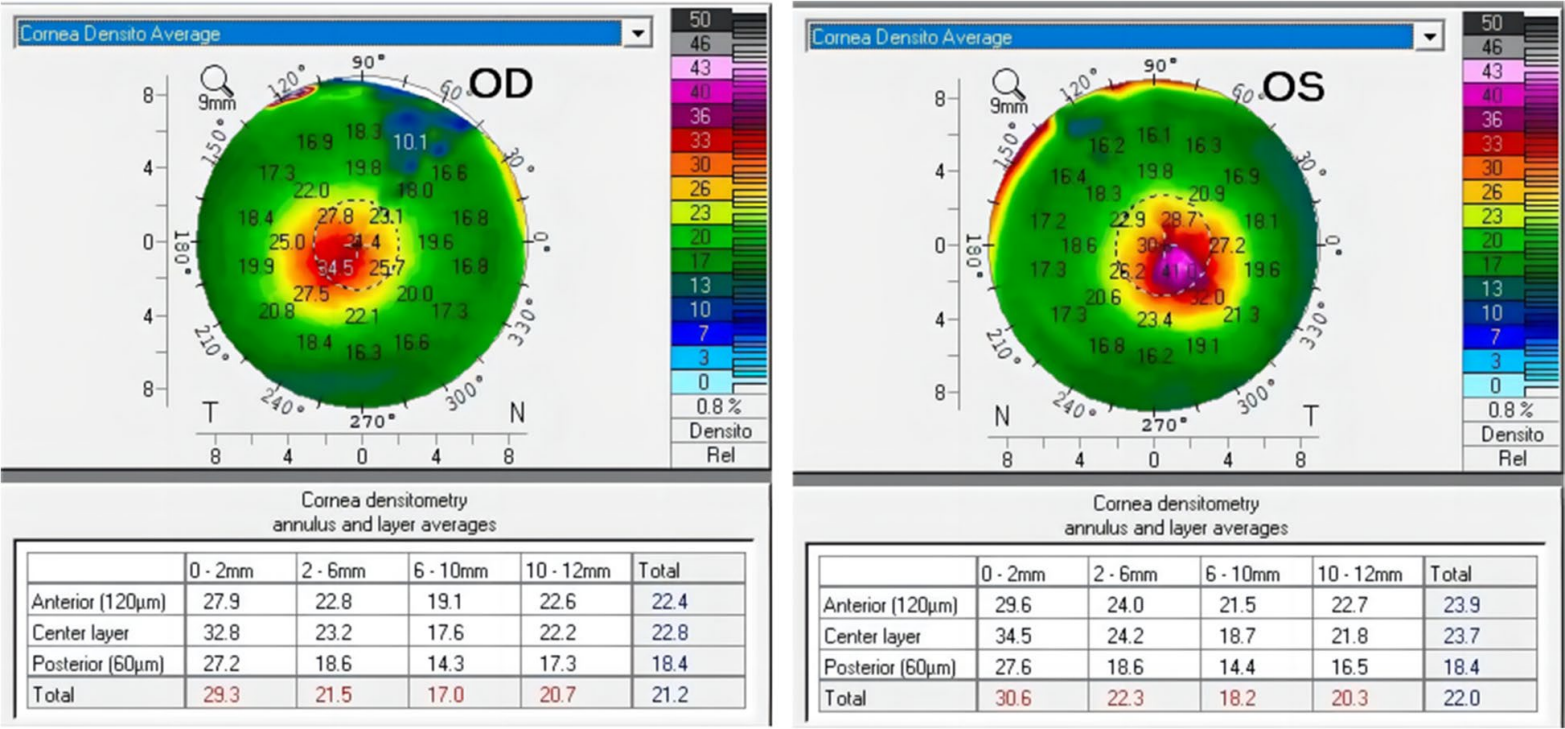
Corneal densitometry of the right and left eyes of case

**Fig. 8:**
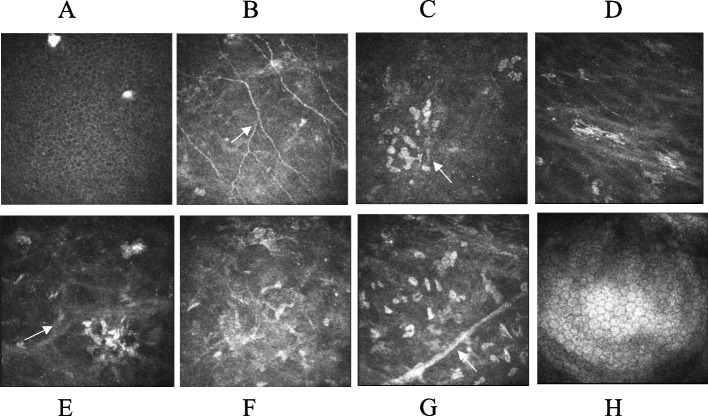
IVCM of the right eye of case: the nerve fibers located beneath the basal cells appeared disordered, as demonstrated by the arrow in  (**B**). Visible were highly reflective fibrotic structures, pointed out by the arrow in  (**C**). The count of stromal cells in the anterior portion of the cornea was significantly diminished, as marked by the arrow in  (**E**). Furthermore, it was challenging to distinguish the cell structure. Additional reflective fibrotic structures were apparent, and notably, the corneal stromal nerves were considerably thicker than average, as indicated by the arrow in  (**G**). The nuclei of the posterior stromal cells varied in size, and no evident activation state was observed

A 36-year-old Chinese female presented to the ophthalmology department of Xinjiang 474 Hospital in May 2020. She was born to parents of fourth-degree consanguineous marriage. The patient's parents, siblings, and two sons all underwent tests including corneal topography, optometry, and a general physical examination, all of which showed no abnormal findings (Fig. 1). The patient had severe osteoporosis, scoliosis of approximately 20° (Fig. 2), hyperextensibility of the joints (Fig. 3), anti-thyroglobulin antibodies, and an elevated parathyroid hormone level. The best corrected visual acuity of the right eye was 14/20, with a manifest refraction of 4.50/-3.75 × 5. The best corrected visual acuity in the left eye was 12/20 with a manifest refraction of -5.25/-3.50 × 5. Intraocular pressure was found to be within normal levels (13 mmHg in both eyes). Slit lamp microscopy showed horizontal nystagmus in both eyes, significant bluish discoloration of the sclera, with decreased central corneal transparency (Fig. 4). High-definition optical coherence tomography (HD-OCT) showed thinning of the entire cornea as well as a heterogeneous and mildly elevated stromal layer density (Fig. 5). The posterior segment showed normal characteristics of the retina. Pentacam anterior segment tomography showed increased corneal curvature in both eyes, with a maximum keratometric power of 50.5 D in the right eye and 50.60 D in the left eye. The thinnest point of each of the patient’s corneas was assessed by Pentacam, which showed that the right eye had a minimum corneal thickness of 285 μm with astigmatism (-4.4D at 174 degrees) and the left eye had a minimum corneal thickness of 280 μm with astigmatism (-3.9 D at 11 degrees), with a white to white value of 11.6 mm in the right eye and a value of 11.7 mm in the left eye (Fig. 6). Corneal densitometry results showed a significant increase in optical density in the 2-mm central area of the cornea. The values for the right and left eye were 29.3 grayscale units (GSU) and 30.6 GSU, respectively (Fig. 7). IVCM results showed that corneal epithelial cell morphology in the central cornea zone was disturbed. Furthermore, nerve fibers under the basal cells were found to be disordered with visible and highly reflective fibrotic structures. The number of stromal cells in the anterior part of the cornea was significantly reduced, their cell structure was difficult to distinguish, and they had visible highly reflective fibrotic structures. Furthermore, the corneal stromal nerves were found to be thicker than average; with the nuclei of the posterior stromal cells showing uneven size distribution with no apparent activation state (Fig. 8). Axial length values were determined to be 21.25 mm in the right eye and 21.13 mm in the left eye, respectively. Utilizing high-throughput sequencing, a homozygous mutation in PRDM5; NM_018699.2:c.247C > T(p.Arg83Cys) was detected. Therefore, the patient can be definitively diagnosed with BCS (OMIM:614,170). Since the patient had fair vision, we educated the patient about safety precautions and asked her to come for follow-up visits every 3 to 6 months. Fortunately, there was no significant decrease in the patient's best-corrected visual acuity, corneal topography curvature, corneal thickness, or corneal densitometry during the two-year follow-up period.

## Discussion and conclusion

Currently, there are no clear diagnostic criteria for BCS in the clinic. Therefore, early genetic detection is necessary for the diagnosis of BCS. Previous studies have shown that BCS originates from mutations in one of two genes: ZNF469 (encoding zinc finger protein 469) and PRDM5 (encoding PR domain-containing protein 5) [4]. According to previous studies and in association with the genes mentioned above, patients with BCS exhibit several standard clinical features. For example, ocular features include extreme corneal thinning, blue sclera, keratoconus, keratoglobus, and high myopia. Extraocular manifestations include deafness, scoliosis, slight joint hypermobility, and developmental dysplasia of the hip. Consanguineous parentage is a significant risk factor for development of BCS. This patient had most of these typical extraocular features. A study by Burkitt et al. [3] concluded that central corneal thickness at approximately or below 400 μm is a potentially reliable diagnostic indicator for BCS. Furthermore, the patient in this study had nystagmus, which was explored in a prior study by Micheal et al. [5] in which they investigated the genetic cause of BCS in a large, consanguineous Pakistani family with four affected individuals. Two of these individuals had nystagmus. The inclusion of BCS in the revised 2017 international Ehlers-Danlos Syndrome (EDS) classification emphasizes its connection with EDS [6, 7]. In situations where a corneal rupture has not yet occurred, it can often be confused with other connective tissue diseases, such as Marfan syndrome [8, 9], osteogenesis imperfecta (OI) [10, 11], or kyphoscoliotic Ehlers-danlos syndrome (kEDS) [12–14]. Given the overlapping clinical presentations and pathophysiological features of these diseases, accurate identification and diagnosis in the early stages can often be challenging. Genetic testing can play a crucial role in the diagnosis of these conditions.

To our knowledge, no studies thus far have used IVCM methods to detect BCS in the clinic, probably because most BCS cases are diagnosed after corneal rupture. The thinness and fragility of the cornea in BCS as well as the small number of diagnosed cases have contributed to the need for doctors to use this method to better identify the disease. There are currently more studies using IVCM to observe keratoconus. Several studies have shown that cell densities in the corneal epithelium such as anterior stromal keratocytes and posterior stromal keratocytes are reduced in patients with keratoconus. Furthermore, decreased nerve fiber density, thickened sub-basal nerves, and abnormal nerve paths have also been observed in patients with keratoconus [15, 16]. IVCM results from this study showed that this BCS patient had keratoconus. In addition, the morphology of the corneal epithelial cells in the central region of the cornea was disordered and irregularly rounded. Furthermore, the basement membrane plexus was found to have a highly reflective fibrotic structure, while the corneal anterior stroma had reduced transparency and was found to be highly reflective and fibrotic. The patient had a significantly reduced number of stromal cells with indistinguishable cell structures. The posterior stromal cell nuclei were not uniform in size and did not show any apparent activation.

In recent years, corneal densitometry has been extensively studied in keratoconus [17–20]. Corneal densitometry has been proposed as a tool for identifying keratoconus [21], and may also be useful in tracking its progression. It's been suggested that the severity of keratoconus [20] is directly associated with the extent of densitometry. Anayol et al. [19] found that corneal densitometry was higher in the anterior stroma 0–2 mm and 2–6 mm areas in keratoconus than in normal populations; Shen et al. [20] concluded that the central 0–2 mm and 2–6 mm anterior layer of the keratoconus corneal densitometry was significantly correlated with maximum keratometry and thinnest corneal thickness. The severity of keratoconus may be correlated with the elevation of the corneal densitometry values, especially in the anterior layer. Collagen scarring in keratoconus may occur first in the anterior corneal layer of the central corneal region. This is consistent with the histopathological alteration of keratoconus, which leads to the irregular arrangement of collagen fibers in the corneal stroma. Corneal densitometry could potentially provide more data for reference in the case of BCS patients, and could alert physicians and researchers to pay more attention to this aspect of information. In our patient, the densitometry of the anterior, middle, and posterior layers of the cornea is increased in the central 2 mm of the cornea, probably because the patient's cornea was too thin, making the collagen scarring of the corneal stroma appear to occur in the whole layer, a finding that distinguishes it from keratoconus. Corneal densitometry, which does not touch the cornea, is safer and sharper for follow-up of patients with BCS and can be a crucial objective indicator for assessing the severity of BCS.

Given the high risk of poor visual outcomes after corneal rupture, identifying this disease before rupture occurs is a critical step in protecting patients' function and quality of life. We informed the patient of the risks of the disease. We informed the patient that the latest study currently allows for a modified collagen crosslinking [22] procedure as an intervention option, but the effectiveness must be verified over time. The patient opted for a close follow-up, where we focused on using Pentcam to monitor the patient's corneal curvature and density. Fortunately, the patient showed no significant progress during the two-year follow-up.

IVCM can help us understand the development of corneogenesis in BCS, but there is still some risk for BCS patients when this device touches the cornea. Corneal densitometry is a safe and effective indicator that can not be ignored to evaluate the severity of BCS.

In conclusion, our research highlights the novel utilization of IVCM and corneal densitometry in the evaluation of BCS, a technique not previously reported in the literature. These diagnostic tools offer invaluable insights that deepen our understanding of BCS. Additionally, our research highlights an unusual case of an adult BCS patient presenting with preserved vision, an intact cornea, and nystagmus. This rare finding underlines the importance of timely diagnosis, early intervention, and rigorous follow-up in the management of BCS.

## Data Availability

All data generated or analysed during this study are included in this published article.

## Abbreviations

BCS: Brittle cornea syndrome
IVCM: In vivo confocal microscopy
HD-OCT: High definition optical coherence tomography
GSU: Grayscale units
ZNF469: Encoding zinc finger protein 469
PRDM5: Encoding PR domain-containing protein 5
OI: Osteogenesis imperfect
EDS: Ehlers-danlos syndrome
kEDS: Kyphoscoliotic Ehlers-danlos syndrome
